# Exploring transcriptomic databases: unraveling circadian gene disruptions in lower grade glioma

**DOI:** 10.1038/s41598-024-67559-9

**Published:** 2024-07-23

**Authors:** Weiyu Hou, Weiming Hou, Xueming Zhao

**Affiliations:** 1 Department of Neurosurgery, The First Hospital of Shanxi Medical University, Taiyuan, 030012 China; 2 Department of Medical Engineering, Air Force Medical Center, PLA, Beijing, 100142 China

**Keywords:** Circadian rhythm, Lower grade glioma, Immune cell infiltration, Survival analysis, DNA methylation, Biomarkers, Genetics research, Translational research, Cancer

## Abstract

The study explored the role of circadian rhythm genes (CRGs) in lower grade glioma (LGG) development and found that certain genes, such as *CRY1, NPAS2,* and *RORB*, were associated with increased or decreased risk of LGG. The study also investigated the correlation between CRGs and immune cell infiltration, revealing a negative association with macrophage infiltration and a positive correlation with B cell and CD8 + T cell infiltration. Additionally, the study identified major mutated CRGs, including *PER2, BMAL1, CLOCK,* and *BMAL2*, and their potential interaction with other CNS-associated genes. The study suggests that CRGs play a crucial role in immune response and tumorigenesis in LGG patients and warrants further investigation.

## Introduction

One kind of cancer that starts in the brain's glial cells is called glioma. Lower grade gliomas (LGG), which include grades II and III, were investigated by TCGA. They can be due to some genetic mutations or environmental factors that are responsible for the growth of these tumors. There are no known causes of gliomas, and the risk factors favoring the development are poorly understood^1^.

The circadian rhythm genes (CRGs) regulate physiological processes such as sleep, metabolism, immune function, and cell cycle in the human body^2^. Brain tumor is a genetic-related disease, and a recent research has found that CRGs may play a crucial role in the occurrence and progression of brain tumors^3^. Patients with brain tumors may experience disruptions in their circadian rhythm, manifested as sleep disorders, abnormal mental states, and disturbances in activity patterns. The investigation of the correlation between brain tumors and CRGs aids in comprehending how disturbances in the circadian rhythm impact the disease progression and quality of life of individuals with brain tumors.

The CRGs play a crucial role in drug metabolism and drug responsiveness^4^. By studying the variations in these genes among brain tumor patients, treatment strategies can be optimized through personalized administration timing and dosage, ultimately leading to improved therapeutic outcomes. CRGs may have potential value in the prognosis evaluation and prediction of brain tumor patients^5^. By analyzing the association between CRGs and patient survival rates, treatment response, and risk of recurrence, more accurate prognostic indicators and personalized treatment recommendations can be provided to clinicians.

## Materials and methods

### Study design

This study aimed to explore the relationship between core CRGs and various aspects of brain tumor biology. Multiple datasets, including gene expression, methylation, mutation, and clinical data from brain tumor patients, were utilized to conduct comprehensive analyses.

The study began by examining the expression levels of CRGs in brain tumors. Gene expression data from tumor samples was analyzed and correlated with patient clinical data. Survival analyses were performed to assess the association between gene expression levels and patient survival. Next, the study investigated the correlation between CRGs and immune cell infiltration in brain tumors. RNA sequencing data was utilized for this analysis. Through deconvolution analysis, we estimated the presence and abundance of immune cell populations within the tumor microenvironment. Additionally, the study explored the relationship between CRGs methylation levels and transcriptional regulation. By analyzing DNA methylation and gene expression data from brain tumor samples, we identified potential associations and correlations. To further investigate causal relationships, Mendelian randomization was employed. This allowed us to explore the potential causal effects of multiple genes on brain tumor development and immune cell infiltration.

Overall, our study utilized a multidimensional approach, integrating various datasets and conducting extensive statistical and bioinformatics analyses. By investigating the expression, methylation, and causal effects of CRGs, as well as their correlations with immune cell infiltration, we aimed to provide insights into the complex biology of brain tumors.

### Data source

The expression data of CRGs in LGG patients and control group with was downloaded in *Gene Expression Profiling Interactive Analysis (GEPIA)* (http://gepia.cancer-pku.cn/) and *cBioPortal for Cancer Genomics* (https://www.cbioportal.org). Immunity infiltration data was downloaded in *TIMER2.0* database (http://timer.cistrome.org/). Circadian gene information was collected from *Circadian Gene DataBase (CGBD)* (http://cgdb.biocuckoo.org/).

### Statistical analysis

Most correlation analysis, differential analysis, immune cell infiltration and survival analysis are based on online database *GEPIA2.0*^6^ (http://gepia2.cancer-pku.cn/#index), *TIMER2.0*^7–9^ and *cBioPortal*^10–12^. Mendelian randomization (MR) study is based on the online platform *MR-base* (http://app.mrbase.org/)^13,14^. When we did MR studies, we set p-value threshold for exposure selection as 5e-08. Linkage Disequilibrium R-square (LD Rsq) setting was 0.001 to exclude LD. Clumping distance was 10,000 kb for clumping analysis.

We used online platform *Evenn* (http://www.ehbio.com/test/venn/#/) to make venn graphs^15^. Other statistical analyses are based on *R* (Version 4.3.1).

## Results

### CRGs expression level in LGG

**Figure 1:**
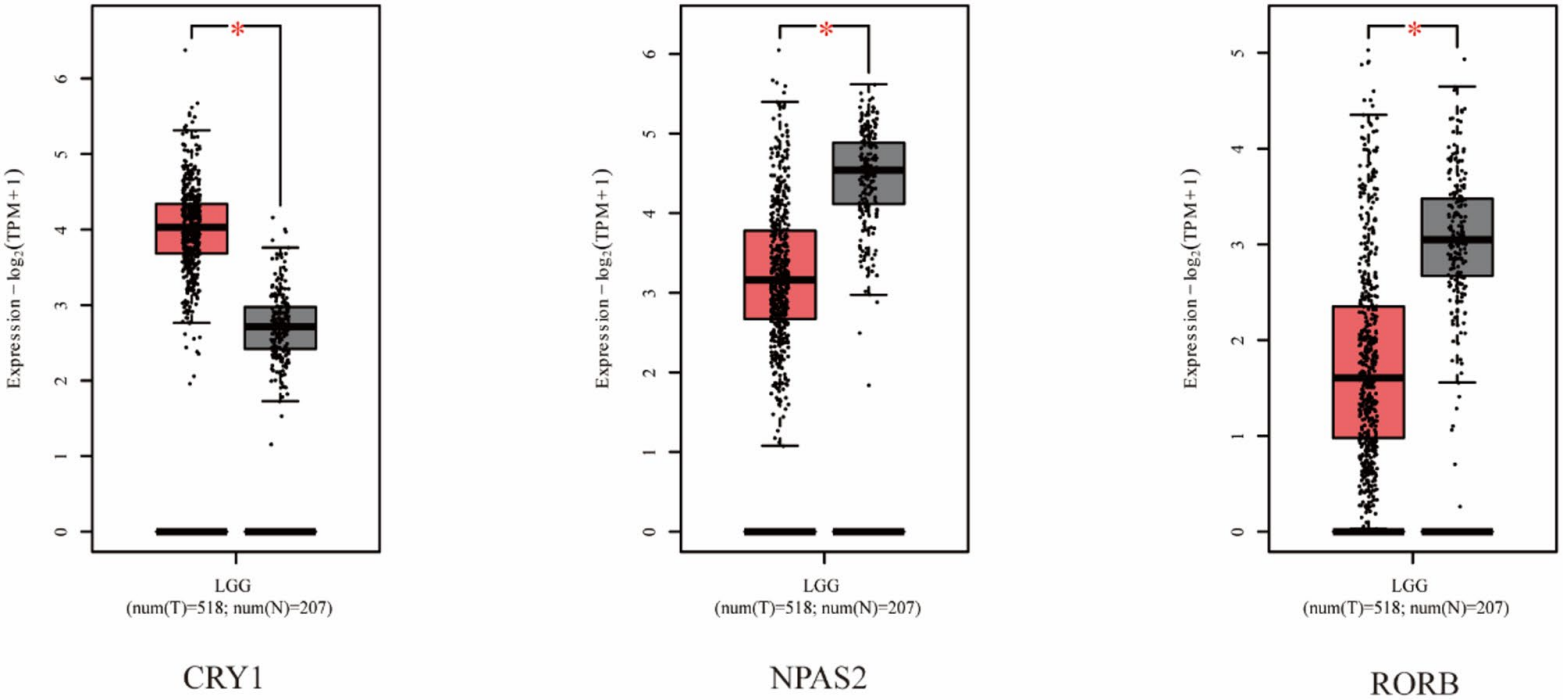
Circadian gene expression in LGG (red boxes) compared to normal brain samples (grey boxes); *TPM* Transcripts Per Million; Significance level is 0.01.

15 core CRGs were identified, with 7 showing high expression (*CRY1, CRY2, NR1D2, PER1, PER2, PER3, RORA*) and 5 showing low expression (*BMAL1/ARNTL, BMAL2/ARNTL2, NPAS2, NR1D1, RORB*)*. CRY1* was significantly upregulated, while *NPAS2* and *RORB* were significantly downregulated (Fig. 1). *CLOCK, NR1D2, PER1, PER2, PER3,* and *RORA* showed relatively high expression, while *ARNTL, ARNTL2,* and *NR1D1* showed relatively low expression (Figure S1).

### Survival analysis based on gene expression levels of CRGs in LGG

**Figure 2:**
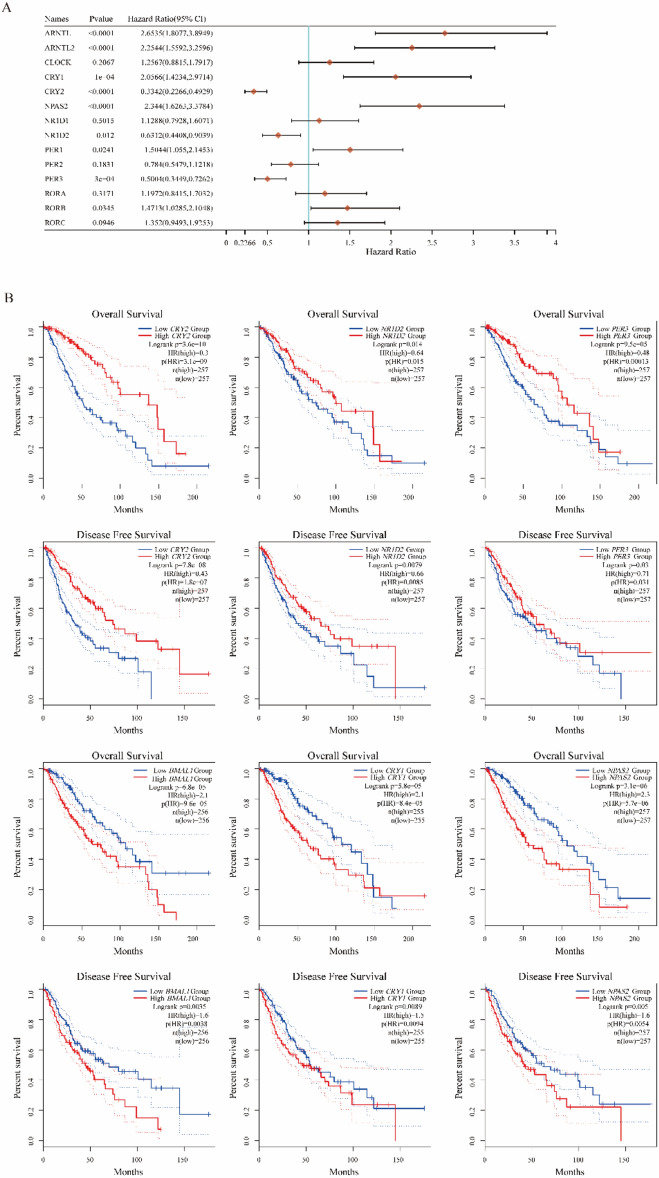
Risks of various CRGs in LGG. (**A**) Hazard ratio (HR) of core circadian genes in LGG. (**B**) Survival analysis of *CRY2, NR1D2, PER3, BMAL1 (ARNTL1), CRY1* and *NPAS2* in LGG by months.

Genes including *CRY2, NR1D2,* and *PER3*, were found to have a positive correlation with high overall survival (OS) and disease-free survival (DFS) in brain tumor patients. They were proved to be the hinder to LGG development (Hazard Ratio (HR) < 1, P < 0.05) (Fig. 2A, B). Specifically, among low and high NR1D2 group, the survival difference is significant (p value = 0.012; HR = 0.6312). Conversely, genes including *ARNTL, ARNTL2, CRY1, NPAS2, PER1,* and *RORB*, were demonstrated to promote the LGG (HR > 1, P < 0.05) (Fig. 2A) and some of them (*ARNTL, CRY1, NPAS2*) showed a negative correlation with OS and DFS (Fig. 2B). Specifically, low expression of *ARNTL*, *CRY1,* and *NPAS2* was associated with high OS and DFS, while high expression of *CRY2, NR1D2,* and *PER3* was associated with high OS and DFS (Figure S2). Notably, ARNTL1 shows significant survival difference (p value < 0.0001; HR = 2.6535). Additionally, in the survival curves, there is an intersection observed of two Kaplan–Meier (KM) curves for *ARNTL2, NR1D1, RORA,* and *RORC* (Figure S2).

### Immune cell infiltration levels

It has been discussed that EPIC and QUANTISEQ results should be recommended as a routine computational method used to assess immune cell infiltration in tumors^16^. We selected EPIC results about B cell, CD4 + T cell, CD8 + T cell and Macrophage, QUANTISEQ result from Myeloid dendritic cell and CIBERSORT result from Neutrophil. As shown in Table 1, the expression of most CRGs showed a negative correlation with macrophage infiltration. *PER1* showed an opposite trend compared to other CRGs. The expression of most CRGs had a strong positive correlation with B cell and CD8 + T cell infiltration, followed by CD4 + T cells and myeloid dendritic cells. The correlation between the expression of most CRGs and neutrophil infiltration was not significant, except for *CLOCK, CRY2,* and *NR1D1*.

**Table 1:** Immune cell infiltration of circadian genes.

Gene	B cell_EPIC			T cell CD4 + _EPIC			T cell CD8 + _EPIC		
	Correlation	P value	Adjusted P value	Correlation	P value	Adjusted P value	Correlation	P value	Adjusted P value
ARNTL	0.240	0.000	0.000	0.248	0.000	0.000	0.282	0.000	0.000
ARNTL2	0.307	0.000	0.000	0.350	0.000	0.000	0.441	0.000	0.000
CLOCK	0.476	0.000	0.000	0.455	0.000	0.000	0.462	0.000	0.000
CRY1	0.121	0.008	0.034	0.131	0.004	0.016	0.157	0.001	0.004
CRY2	0.603	0.000	0.000	0.554	0.000	0.000	0.484	0.000	0.000
NPAS2	0.326	0.000	0.000	0.376	0.000	0.000	0.416	0.000	0.000
NR1D1	0.382	0.000	0.000	0.330	0.000	0.000	0.282	0.000	0.000
NR1D2	0.570	0.000	0.000	0.509	0.000	0.000	0.492	0.000	0.000
PER1	− 0.153	0.001	0.010	− 0.169	0.000	0.002	− 0.115	0.012	0.074
PER2	0.411	0.000	0.000	0.398	0.000	0.000	0.388	0.000	0.000
PER3	0.244	0.000	0.000	0.116	0.011	0.036	0.107	0.019	0.085
RORA	0.402	0.000	0.000	0.326	0.000	0.000	0.357	0.000	0.000
RORB	0.485	0.000	0.000	0.456	0.000	0.000	0.493	0.000	0.000
RORC	0.121	0.008	0.045	0.108	0.018	0.066	0.219	0.000	0.000

Gene	Myeloid dendritic cell_QUANTISEQ			Macrophage_EPIC			Neutrophil_CIBERSORT		
	Correlation	P value	Adjusted P value	Correlation	P value	Adjusted P value	Correlation	P value	Adjusted P value
ARNTL	0.149	0.001	0.007	− 0.182	0.000	0.000	0.035	0.448	0.572
ARNTL2	0.347	0.000	0.000	− 0.114	0.013	0.028	0.071	0.119	0.179
CLOCK	0.165	0.000	0.002	− 0.143	0.002	0.006	0.127	0.006	0.011
CRY1	0.239	0.000	0.000	− 0.070	0.124	0.222	0.062	0.173	0.277
CRY2	− 0.063	0.171	0.329	− 0.506	0.000	0.000	− 0.132	0.004	0.013
NPAS2	0.320	0.000	0.000	− 0.231	0.000	0.000	− 0.023	0.621	0.750
NR1D1	0.037	0.415	0.676	− 0.297	0.000	0.000	− 0.135	0.003	0.022
NR1D2	0.066	0.149	0.333	− 0.317	0.000	0.000	− 0.031	0.493	0.659
PER1	− 0.199	0.000	0.000	0.032	0.486	0.715	− 0.008	0.870	0.962
PER2	0.217	0.000	0.000	− 0.266	0.000	0.000	− 0.072	0.114	0.228
PER3	− 0.153	0.001	0.005	− 0.081	0.077	0.157	− 0.062	0.174	0.310
RORA	0.108	0.018	0.060	− 0.255	0.000	0.000	0.031	0.502	0.615
RORB	0.170	0.000	0.001	− 0.364	0.000	0.000	− 0.031	0.500	0.675
RORC	0.232	0.000	0.000	− 0.082	0.074	0.193	0.006	0.895	0.949

P value = 0.000 means P value < 0.001.

### CRGs in LGG with gene mutation and correlation

**Figure 3:**
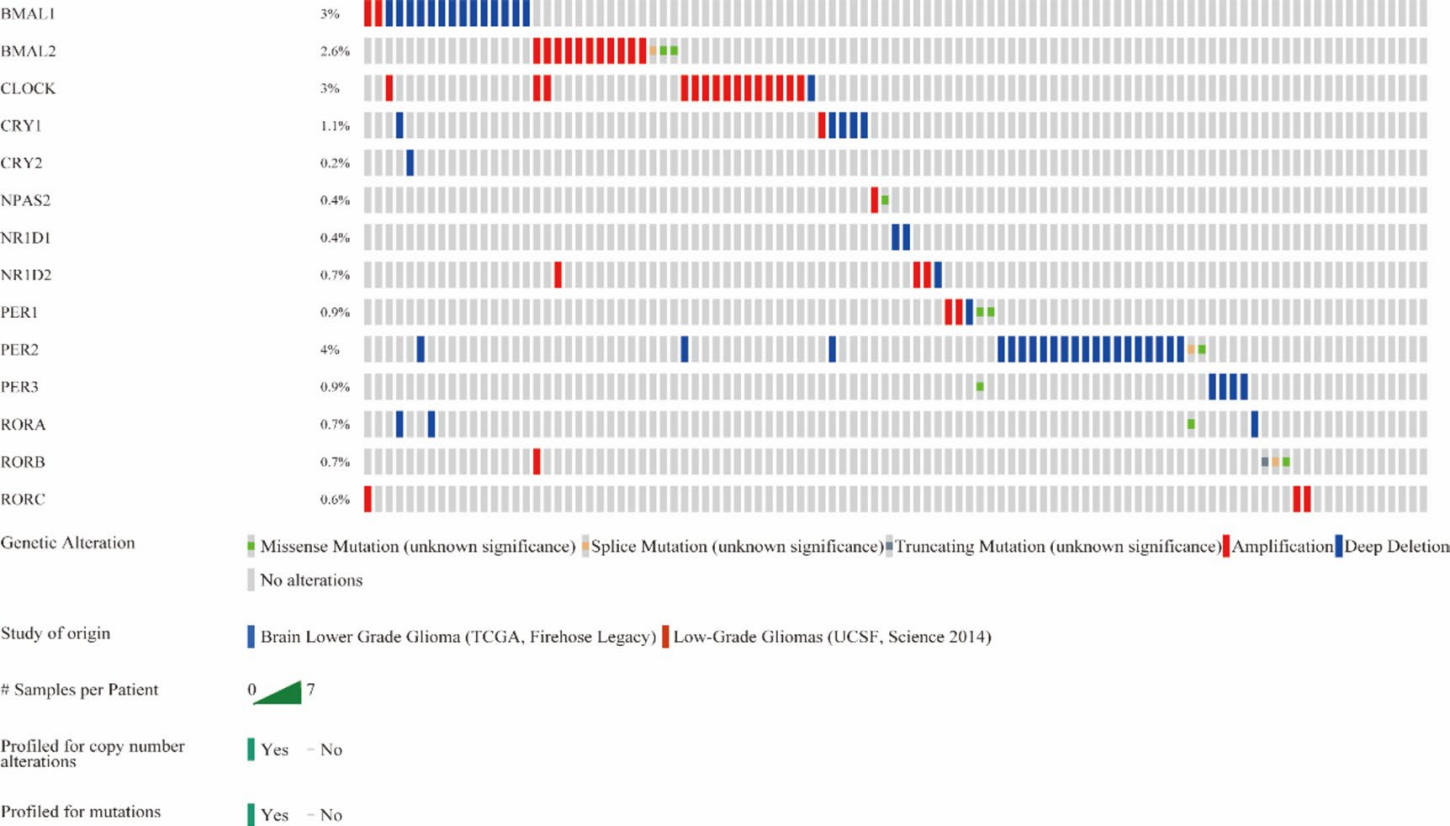
Frequency of mutations in LGG based on CBioPortal.

**Figure 4:**
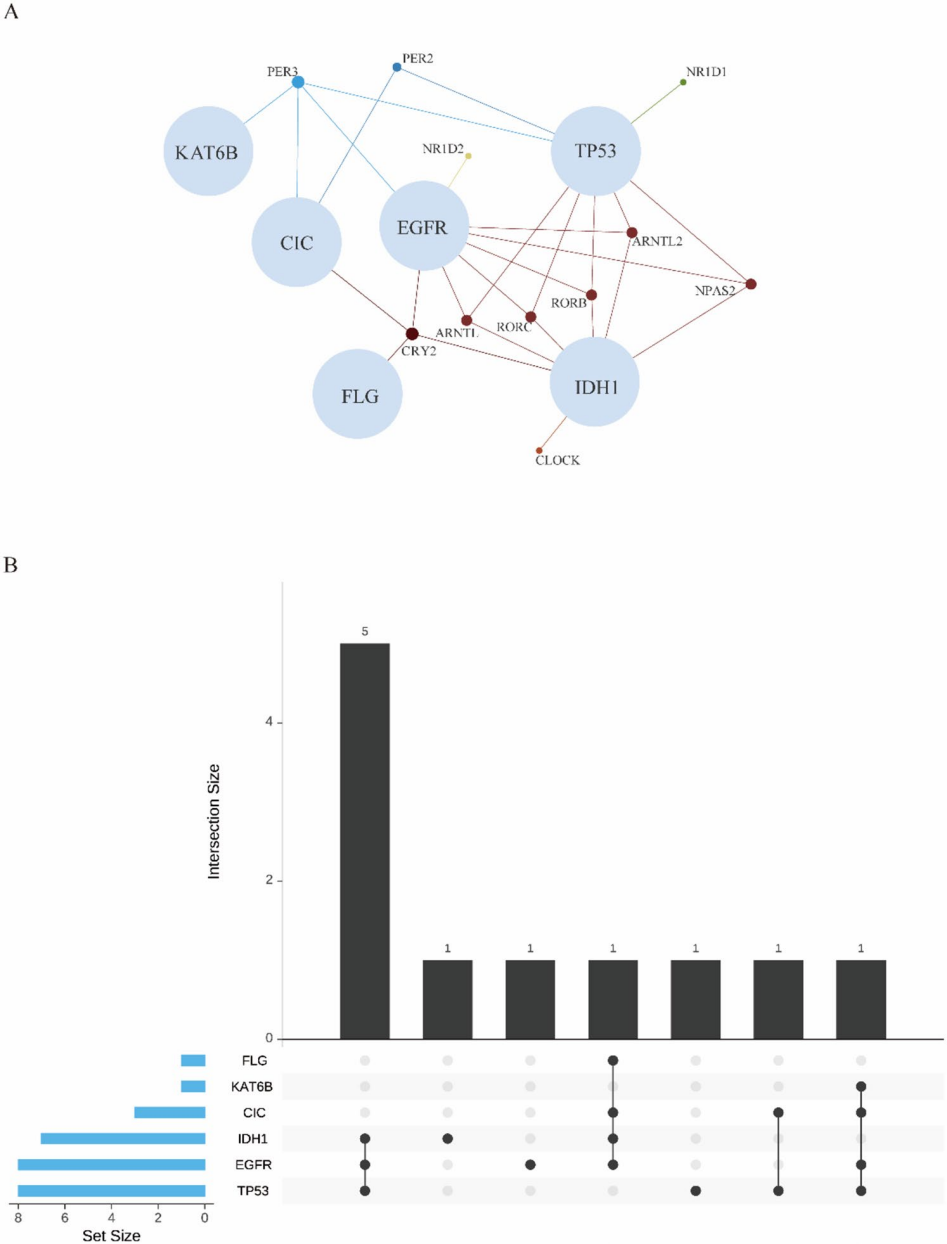
(**A**) Venn network analysis of circadian genes expression in major mutations from LGG. (**B**) Upset plot for different amount of valuable circadian genes among different gene mutations (FLG, KAT6B, CIC, IDH1, EGFR and TP53).

LGG mainly mutated CRGs are *PER2, BMAL1, CLOCK,* and *BMAL2* (Fig. 3). According to the recent study, the significantly mutated genes in LGG are *ARIDIA, CIC, EGFR, IDH1, KAT6B, PIK3R1, TP53* and *FLG*^17^. In differential analysis of CRGs expression between mutated and non-mutated groups of the mutated genes above in LGG (Figure S3), we found that *NPAS2* showed significant differential expression between the *ARID1A* mutated group and the non-mutated group. *CRY2, PER2,* and *PER3* displayed significant differential expression between the *CIC* mutated group and the non-mutated group. *ARNTL, ARNTL2, CRY2, NPAS2, NR1D2, PER3, RORB,* and *RORC* exhibited significant differential expression between the *EGFR* mutated group and the non-mutated group. *CRY2* demonstrated significant differential expression between the *FLG* mutated group and the non-mutated group (Wilcoxon, p = 0.017). *ARNTL, ARNTL2, CLOCK, CRY2, NPAS2, RORB,* and *RORC* showed significant differential expression between the *IDH1* mutated group and the non-mutated group. *PER3* displayed significant differential expression between the *KAT6B* mutated group and the non-mutated group (Wilcoxon, p = 0.012). *ARNTL, ARNTL2, NPAS2, NR1D1, PER2, PER3, RORB,* and *RORC* exhibited significant differential expression between the *TP53* mutated group and the non-mutated group. Also, we discovered EGFR, IDH1 and TP53 mutated groups have more altered CRGs via Venn network analysis (Fig. 4A, B).

Then we did the differential gene analysis in immune related genes among CRGs mutation groups. Differential gene expression analysis revealed significant differences between mutated and non-mutated groups for several immune-related genes (Figure S4). Specifically, *AADAT* showed significant differential expression in *ARNTL2* mutated groups (Wilcoxon, p = 0.026), while *AAAS* showed significant differential expression in the same group (Wilcoxon, p = 0.044). In *CREBBP* mutated groups, differential expression was found for *APITD1, APOBEC3B, LAMP3, CXCL9, TIGIT, TNFRSF9, GPNMB, MMP9, PHEX,* and *TAP1*. Furthermore, the *PER3* mutated group exhibited significant differential expression of *NR1D1* (Wilcoxon, p = 0.046), while *CREBBP* mutated groups showed significant differential expression of *ARNTL2* and *CRY1*.

**Figure 5:**
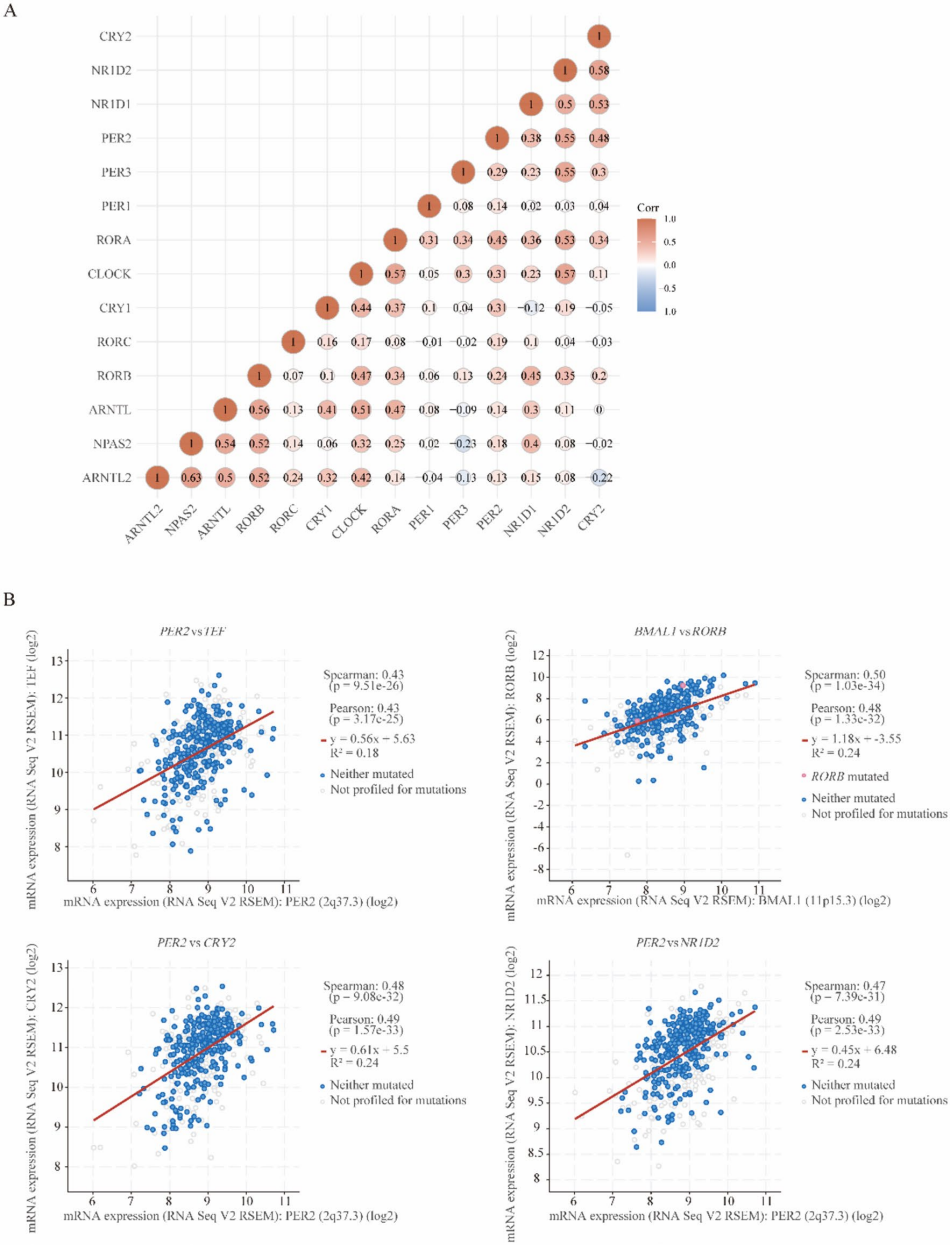
Correlation and Co-Expression Analysis of Circadian Genes in Relation to LGG. (**A**) Correlation analysis between circadian genes. Corr: correlation effect. (**B**) Co-expression analysis of circadian genes by both Spearman and Pearson statistics.

In the correlation analysis of CRGs, significant associations were identified between different genes (Fig. 5A, B). Particularly, a strong positive correlation was observed between *NPAS2* and *ARNTL2*. Additionally, a strong positive correlation was also found between *CRY2* and *NR1D2*. On the other hand, a strong negative correlation was observed between *NPAS2* and *PER3*. Similarly, a strong negative correlation was identified between *CRY2* and *ARNTL2*, further highlighting a potential regulatory interaction between these CRGs.

In the correlation analysis of CRGs and immune-related genes, distinct associations were observed among various gene pairs (Figure S5). Notably, *ARNTL1* exhibited a correlation with *AAAS* (rho =  − 0.128, p = 3.52 × 10^–3^). Furthermore, strong correlations were found between *ARNTL2* and *A2BP1, AAAS, CXCL9, GPNMB, MMP9, PHEX, TAP1, TIGIT,* and *TNFRSF9*. Similarly, *CLOCK* displayed positive correlations with *A2BP1, CXCL9, PHEX, TIGIT,* and *TNFRSF9*. Moreover, *CREBBP* showed significant correlations with *APITD1, APOBEC3B, GPNMB,* and *TNFRSF9*. Additionally, *NPAS2*, *NR1D1*, *PER3*, *RORB*, and *RORC* were positively correlated with *AAAS*.

### CRGs methylation

**Figure 6:**
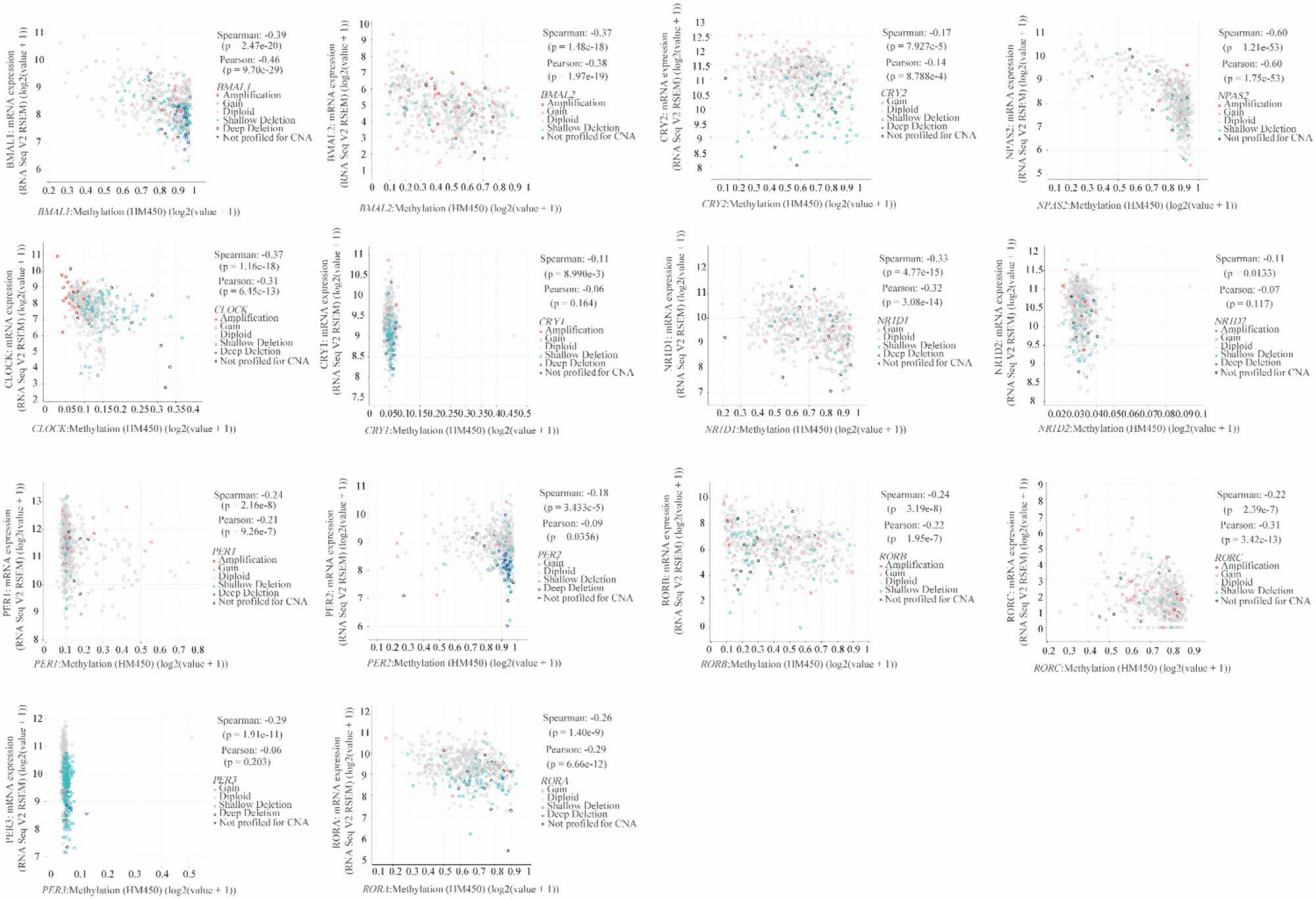
Correlation between mRNA expression of circadian genes and their methylation by both Spearman and Pearson statistics.

We have done correlation analysis between CRGs methlation and their mRNA expression. Methylation of various CRGs showed a significant negative correlation with their mRNA expression levels (Fig. 6). Particularly, *NPAS2* methylation showed a strong negative correlation with its transcription level (Spearman’s rho: − 0.60, p = 1.21 × 10^−53^; Pearson’s r: − 0.60, p = 1.75 × 10^−53^).

### Mendelian randomization (MR) studies

In two-sample MR study (Table 2), CREB-binding protein (*CREBBP*) has a positive causality with benign CNS tumors (Inverse variance weighted beta = 0.2329, p = 0.01911). Besides, TNF receptor superfamily member 11b (*TNFRSF11B*) shows weak positive causality with benign CNS tumors as well (Weighted median beta = 0.1243, p = 0.03215). Apolipoprotein B has a negative causality with neutrophil cell count and sleeping disorders including trouble falling or staying asleep, or sleeping too much. *GPNMB* shows positive causality with CD8 + T cell Absolute Count (Wald ratio beta = 0.2068, p = 0.004226). *CXCL9* has weak negative causality with daytime nap meanwhile its reduction can be explained by brain tumor. All single SNP analysis, method comparison plots, leave-one-out analysis and funnel plots are shown in Figure S6.

**Table 2:** Two-sample mendelian randomization study.

Exposure	Outcome	MR results				
		Method	nsnp	b	se	pval
CREB-binding protein	Benign neoplasm of brain and other parts of central nervous system	Weighted median	15	0.2419	0.1209	0.04532
		Inverse variance weighted	15	0.2329	0.09937	0.01911
Apolipoprotein B levels	Brain tumor	Inverse variance weighted	186	0.2138	0.09349	0.02222
		Weighted mode	186	0.2576	0.1078	0.0179
Daytime nap	Apolipoprotein B	Inverse variance weighted	99	0.461	0.2115	0.02925
Daytime nap	Brain tumor	MR Egger	100	1.275	1.896	0.503
TNF receptor superfamily member 11b	Benign neoplasm of brain and other parts of central nervous system	Weighted median	14	0.1243	0.058	0.03215
		Weighted mode	14	0.1311	0.05443	0.0316
CXCL9 levels	Daytime nap	Weighted median	5	− 0.00551	0.002588	0.03317*
Apolipoprotein B	Trouble falling or staying asleep, or sleeping too much	Inverse variance weighted	2	− 0.3111	0.1519	0.0405
Apolipoprotein B levels	neutrophil cell count	Weighted median	183	− 0.0118	0.005631	0.03606
GPNMB	CD8 + T cell Absolute Count	Wald ratio	1	0.2068	0.07228	0.004226
brain cancer	CXCL9 levels	Wald ratio	1	− 146.8	70.6	0.03759

Heterogeneity Statistics				Horizontal pleiotropy		
Method	Q	Q_df	Q_pval	Egger regression intercept	Standard error	Directionality p-value
Inverse variance weighted	18.71	14	0.1763	0.019	0.043	0.666
Inverse variance weighted	206.2	185	0.1368	0.00086	0.005	0.864
Inverse variance weighted	116.3	98	0.1002	− 0.0091	0.0073	0.217
MR Egger	16.68	12	0.1619	Insufficient data for analysis		
MR Egger	2.555	3	0.4654	0.003	0.005	0.585
Inverse variance weighted	0.7951	1	0.3726	NA	NA	NA
MR Egger	2456	181	0	− 0.0001	0.00078	0.897
MR Egger	2456	181	0	Insufficient data for analysis		
No data available in table				Insufficient data for analysis		

*MR* Mendelian randomization, *nsnp* number of single nucleotide polymorphisms, *b* effect sizes for each SNP, *se* standard error, *pval* p value

*p-value threshold for exposure selection is adjusted to 5 × 10^6^.

What’s more, we have discovered daytime nap reasons apolipoprotein B and apolipoprotein causes the brain tumor (Table 2). However, there is no causality between daytime nap and brain tumor. Thus, this is the complete mediation case.

In multivariate MR study (Table 3), we tried to discover caualities between multiple kinds of instruments affected by circadian rhythm and *CREBBP*. These instruments include: variation in diet, leisure/social activities (Sports club or gym), daytime dozing / sleeping (narcolepsy) and mixture of day and night shifts. No significant results were gained (significance level: 0.05).

**Table 3:** Multivariable mendelian randomization study upon different exposure related to circadian rhythm.

Exposure	ID	Outcome	ID	nsnp	b	se	pval
Variation in diet	ukb-b-2909	CREB-binding protein	prot-a-655	14	0.099573	0.688843	0.885065
Leisure/social activities: Sports club or gym	ukb-b-4000			4	0.291531	1.338374	0.827565
Daytime dozing/sleeping (narcolepsy)	ukb-b-5776			30	− 0.83591	0.535812	0.118739
Mixture of day and night shifts worked: This type of shift pattern was not worked during job	ukb-d-22640_9			0	− 0.26489	0.676828	0.695525

## Discussion

We mainly explored core CRGs in mRNA expression level, survival analysis, and immune cell infiltration levels. We found out *CRY1* is significantly associated with increased risk of LGG. Survival analysis also showed *CRY1* caused lower OS and DFS. *CRY1* has been found to be pro-tumorigenic by promoting DNA repair and cell survival through temporal transcriptional regulation^18^. *CRY1* is hormone-induced in tumors, indicating that circadian hormone might be able to affect LGG prognosis. Also, *NPAS2* and *RORB* were both found to decrease LGG risk. It is suspected that *NPAS2* modulates tumorigenesis through the following two aspects: directly carcinogenic mechanism and interaction with other clock proteins^19,20^. Both *NPAS2* and *RORB* also showed lower OS and DFS in survival analysis. However, *NPAS2* might have bidirectional effects in different tumors and deserve further studies. Likewise, although NR1D2 and PER3 do not show significant differece between tumor and normal tissues, they do show significant correlation with survival conditions. It indicates that the mechanism behind how genes influence tumors is complicated and equally-expressed genes deserve more attention in the future studies.

In survival analysis, in *ARNTL2* group two KM curves got intersected. From 0 to 8 years, the survival rate was higher when the *ARNTL2* expression is higher. It demonstrated that *ARNTL2* is anti-tumor during this time. However, after 9 years, it showed the opposite outcome which has been proved to be a potential oncogene, contributing to immunosuppressive tumor microenvironment^21^. Thus, contradictory results shows the value of continuing to dig up how *ARNTL2* influences LGG.

In immune cell infiltration analysis, we detected the negative association between macrophage infiltration and most CRGs. As we have known, tumor-associated macrophages facilitate tumor proliferation, survival and migration^22^. However, macrophages in tumor micro-environment are categorized into pro-tumorigenic and anti-tumorigenic^23^, thus making the mechanism of different CRGs on LGG through macrophage infiltration more complicated. Particularly, the correlation between the *PER1* gene and immune cell infiltration often exhibits an opposite direction compared to other genes. The downregulation of *PER1* can lead to decreased sensitivity of U343 glioma cells to X-ray irradiation by altering the expression of genes associated with cell cycle arrest and apoptosis, such as *c-myc, P53, p21, cdc2 and cyclineB1*. This is achieved through the activation of the *CHK2-P53* pathway, ultimately resulting in reduced apoptosis^24^. Further studies are needed to figure out how *PER1* inhibits tumor growth via affecting different immune cell infiltration.

Most CRGs correlated strongly with B cell and CD8 + T cell infiltration in LGG. This indicates that most CRGs promote or suppress glioma mediated by both immune cells. No significant correlation with neutrophil infiltration except *CRY2* and *NR1D1* whose high expression has been estimated to high OS and DFS. Altered expression of CRGs including *CRY2, PER2 and BMAL1* has been explored to be explained by reduced neutrophil infiltration in liver^25^. We still has no clue how these genes get modulated by neutrophil infiltration in brain which might uncover the specific function of *CRY2* and *NR1D1* on glioma development.

Major mutated CRGs in LGG are *PER2, BMAL1, CLOCK* and *BMAL2*, which might indicate these genes are most influencial CRGs to glioma development or metastasis. Besides, significant expression difference of most CRGs existed in *TP53, EGFR* and *IDH1* mutated groups. This might imply that CRGs greatly influence LGG patients under those mutation background. In addition, within CRGs mutated groups, some CNS-associated genes like *AADAT* and *AAAS* showed significant differencial results. *AADAT* has been firstly investigated in immune response in meningitis patients^26^. *AAAS* may be involved in normal development of the peripheral and central nervous system^27–29^. Also we have discovered *AAAS* has a significant correlation with many CRGs. There is no clear evidence that both genes are related to LGG but it might hint the interaction between CRGs and these genes thus modulating CNS system via circadian rhythm.

Besides, in mutation differential analysis we have found out many significant results from *CREBBP* within extended core clock network genes while core CRGs got nothing. *CREBBP* is a tumor suppressor, whose decreased expression cooperates with the oncogene *MYCN* to induce malignant brain tumors in mice^30^. In our two-sample MR study, *CREBBP* affected benign brain tumors. We found many immune-related genes (*APITD1、APOBEC3B、LAMP3、CXCL9、TIGIT、TNFRSF9、GPNMB、MMP9、PHEX、TAP1*) expressed differently between *CREBBP*-mutated group and non-mutated group. Notably, MMP might be important to cancer cell morphology and angiogenesis with the extracellular matrix^31^. Some of them are even correlated to each other. In our MR studies, we have found some caualities associated with those immune-related genes. Among them, another TNF receptor superfamily has been proved to cause benign CNS tumors. When TNFRSF is present in a gene collection, it can be a valuable molecular marker, a reliable predictor of survival for GBM patients, and a potential target for cancer immunotherapy^32^. Studies have also shown that immune system matters a lot to tumor development^33,34^. And *CXCL9* modulating daytime nap also builds the bridge between immune response and circadian rhythm. Apolipoprotein B can control sleeping disorders and also neutrophil cell count. In mediation effect analysis, we proved apolipoprotein B completely mediates between daytime nap and brain tumor. This signals that this molecule might be necessary target between circadian rhythm disruption and brain tumor. Also *GPNMB* affects CD8 + T cell. In this way, continued research about *APOBEC3B* and *GPNMB* in immune cell infiltration and circadian rhythm should be carried out. These genes might become potential immune targets to LGG.

Some CRGs have shown strong positive or negative correlation with other CRGs. Moreover, some CRGs mutations have differential expression of some other CRGs. Especially, the negative correlation between *NPAS2* methylation and its transcription levels indicates that the regulation of this gene may be inhibited in LGG. Methylation of *NPAS2* may disrupt the normal functioning of circadian rhythm, leading to aberrant timing of physiological processes. It could potentially play a role in the development and progression of LGG. The interaction among different CRGs in LGG is undoubted and awaits more studies.

Our study does have several limitations. One potential limitation is our research lacks in vitro experiments to support the theoretical research points. We look forward to future studies that can complement and refine our findings. Besides there are studies showing that miRNAs associated with cancer immunity can be a useful prognostic marker, so more non-coding RNA researches are required to improve our results^35,36^.

In summary, CRGs play a crucial role in immune response and tumorigenesis in LGG patients. The bridge between circadian rhythm and glioma is expected to strengthened in the near future.

### Supplementary Information

Supplementary Figures.

## Data Availability

Data from **GEPIA, TIMER 2.0, cBioPortal** and **CGDB** is freely available and we have cited necessary papers as requested.
