# Supplementary material for: Exploring transcriptomic databases: unraveling circadian gene disruptions in lower grade glioma
Source: Sci Rep. 2024 Jul 23;14:16960. doi: 10.1038/s41598-024-67559-9 (PMC11266536; doi:10.1038/s41598-024-67559-9)

**Figure S1** Circadian gene expression in LGG (red boxes) compared to normal brain samples (grey boxes); TPM: Transcripts Per Million.


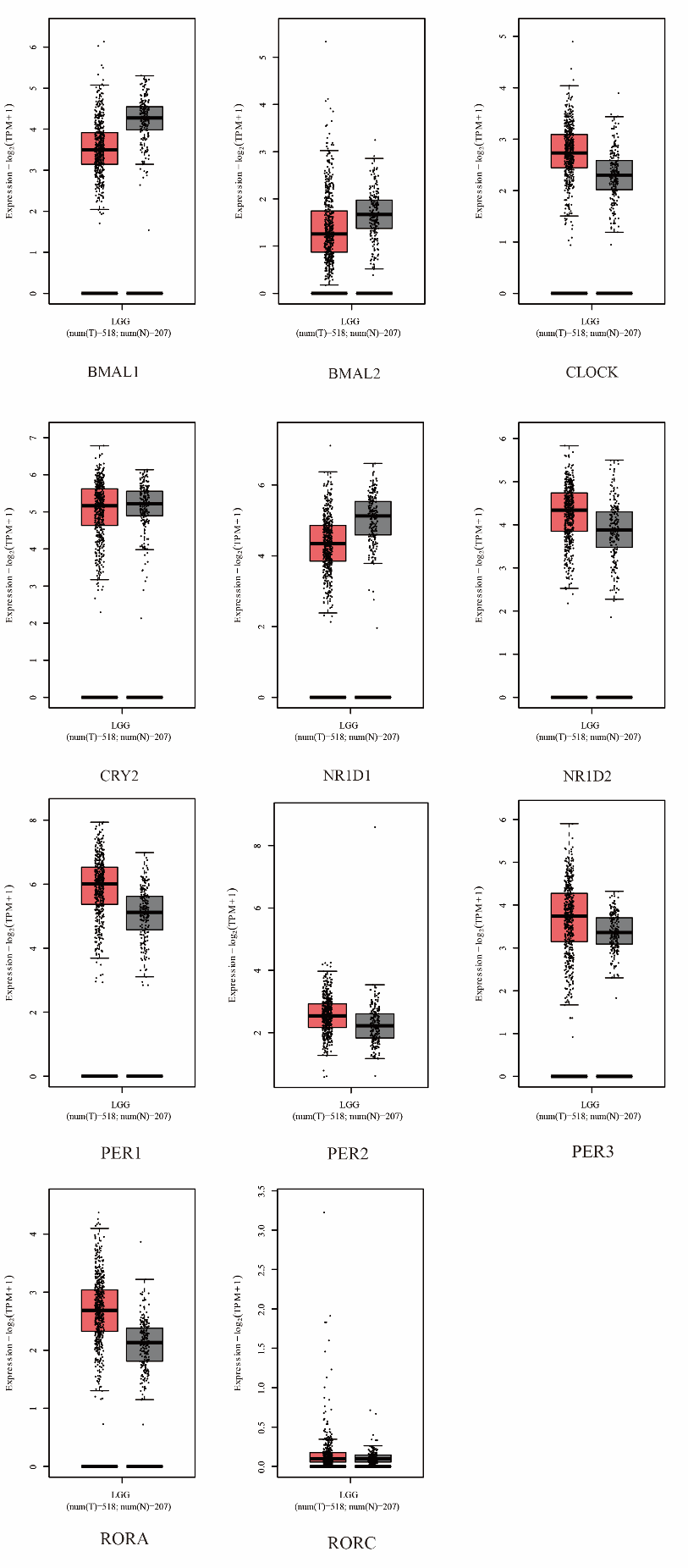


**Figure S2** Survival analysis of other circadian genes in LGG by months.


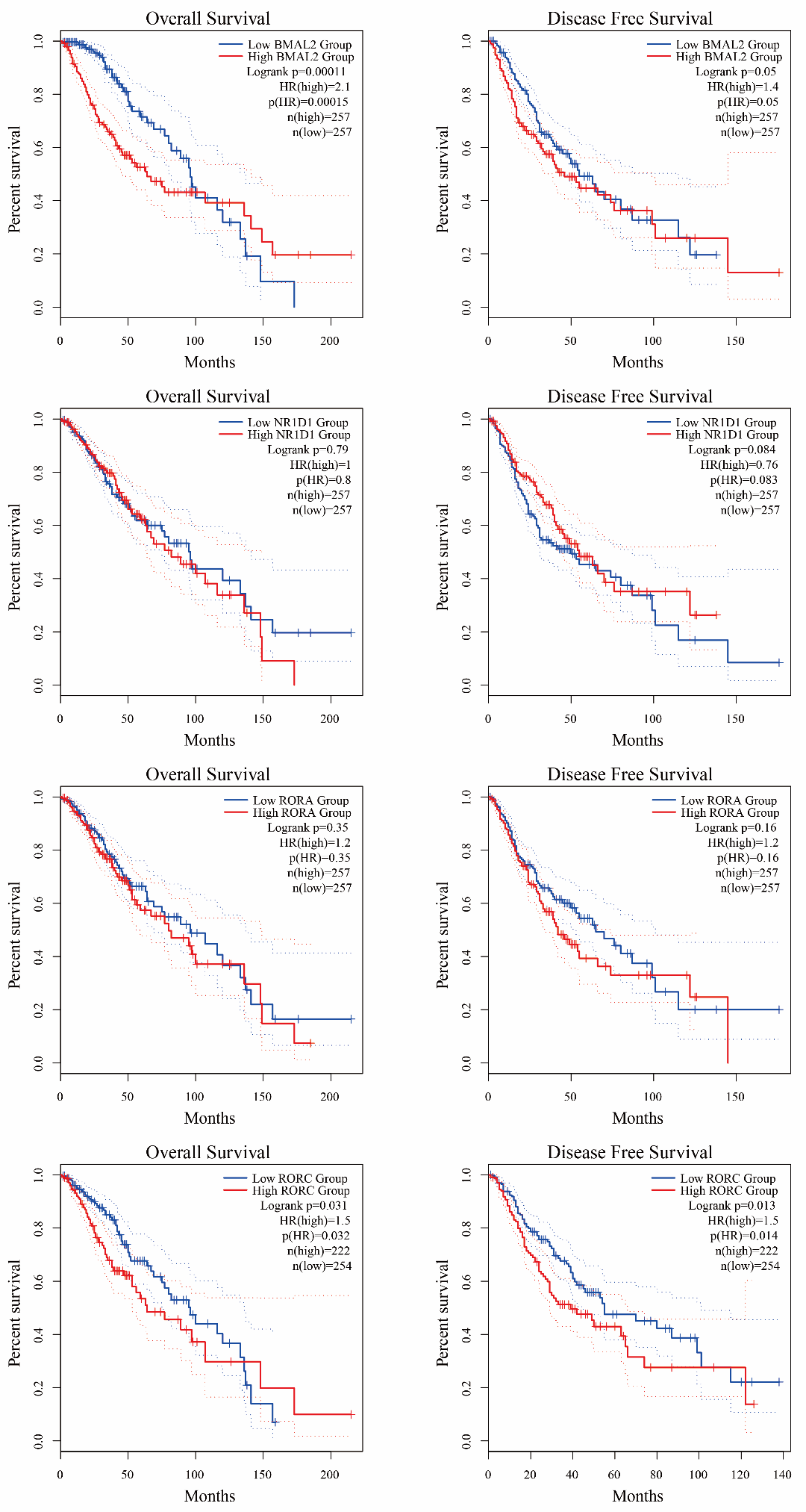


**Figure S3** Differential expression analysis of different genes between ARNTL2, CREBBP and PER3 mutated and non-mutated groups by Wilcoxon test.


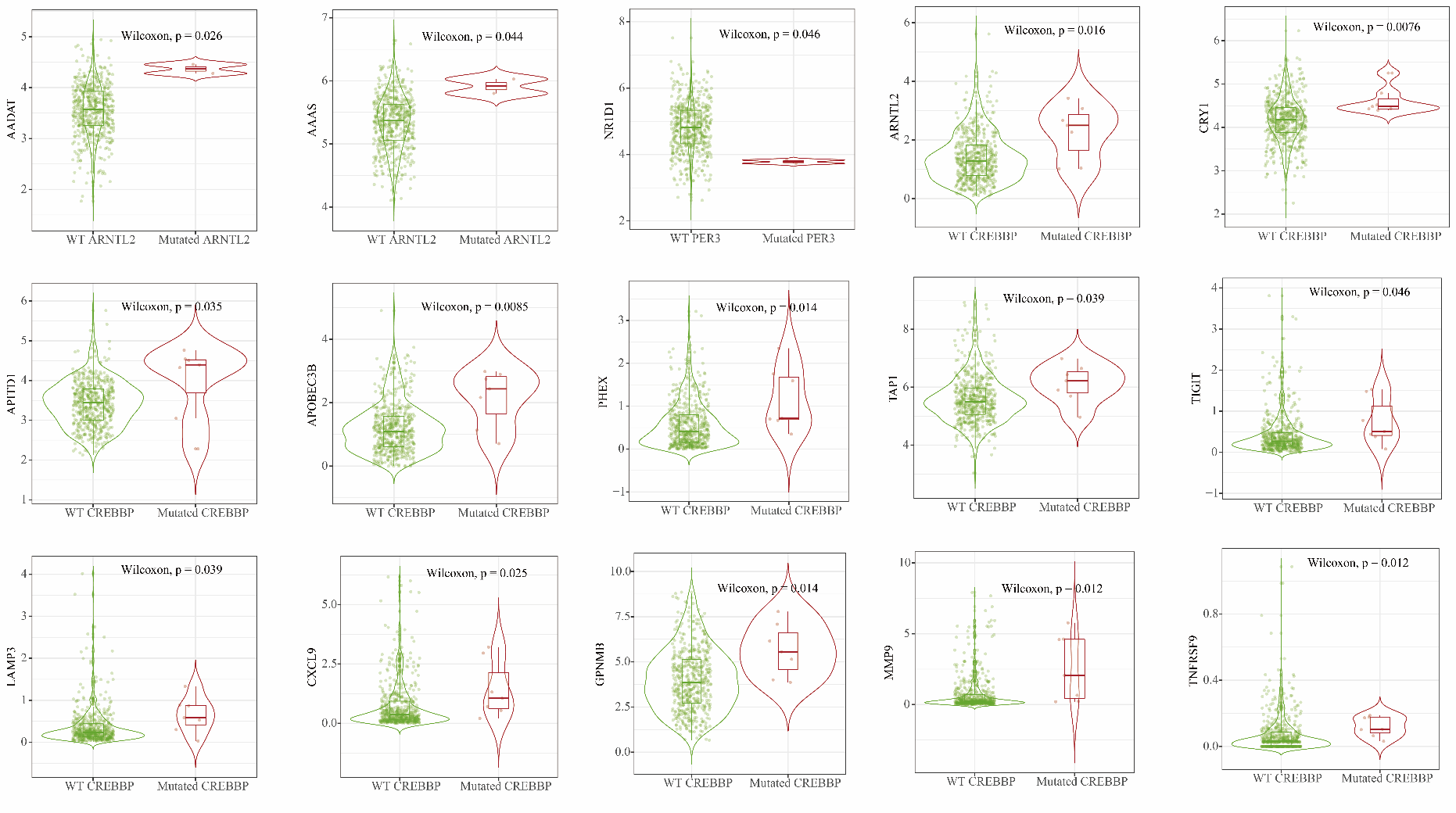


**Figure S4** Differential expression of circadian genes between major mutated genes group in LGG and non-mutated group.


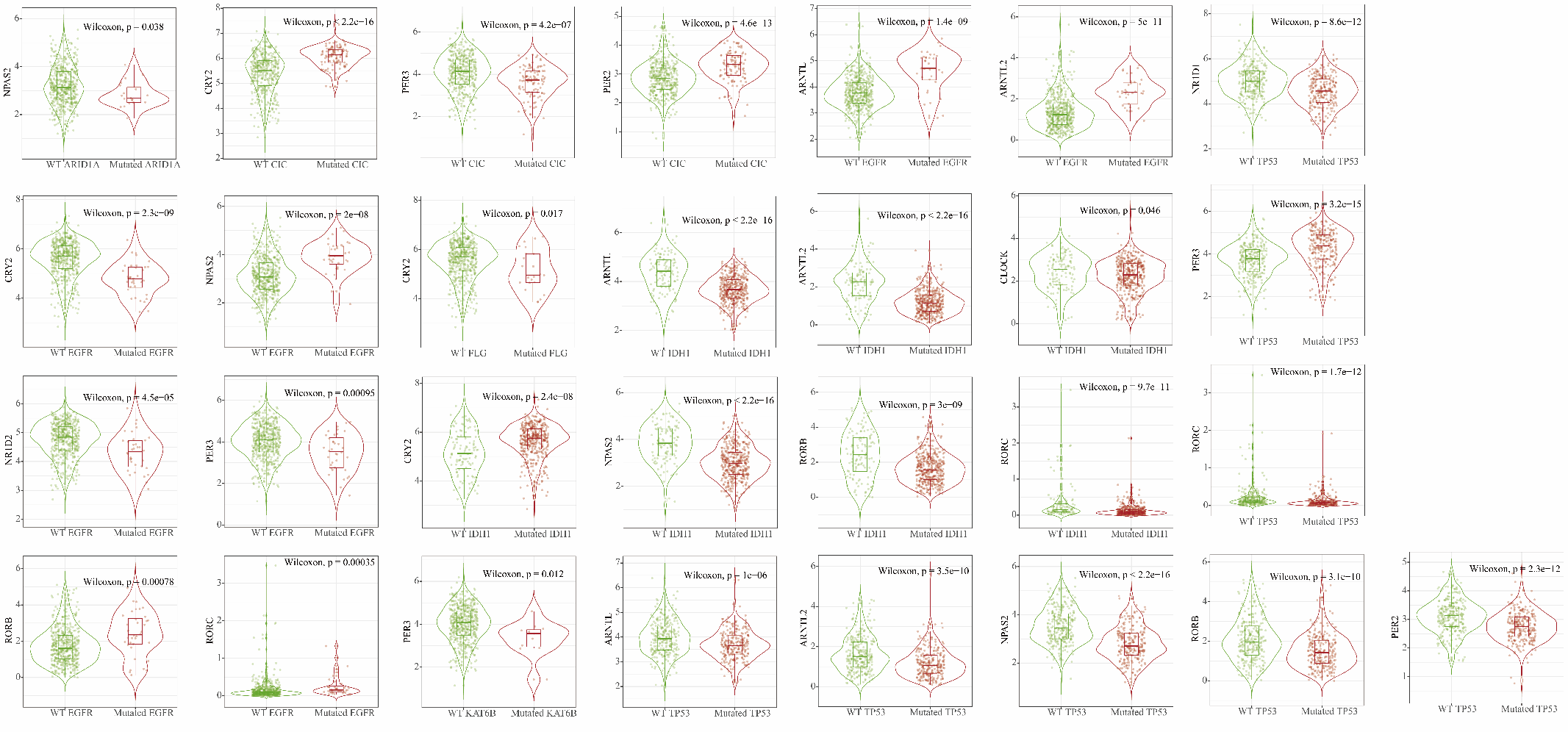


**Figure S5** Co-expression analysis of other circadian genes with immune-related genes by Pearson statistics.


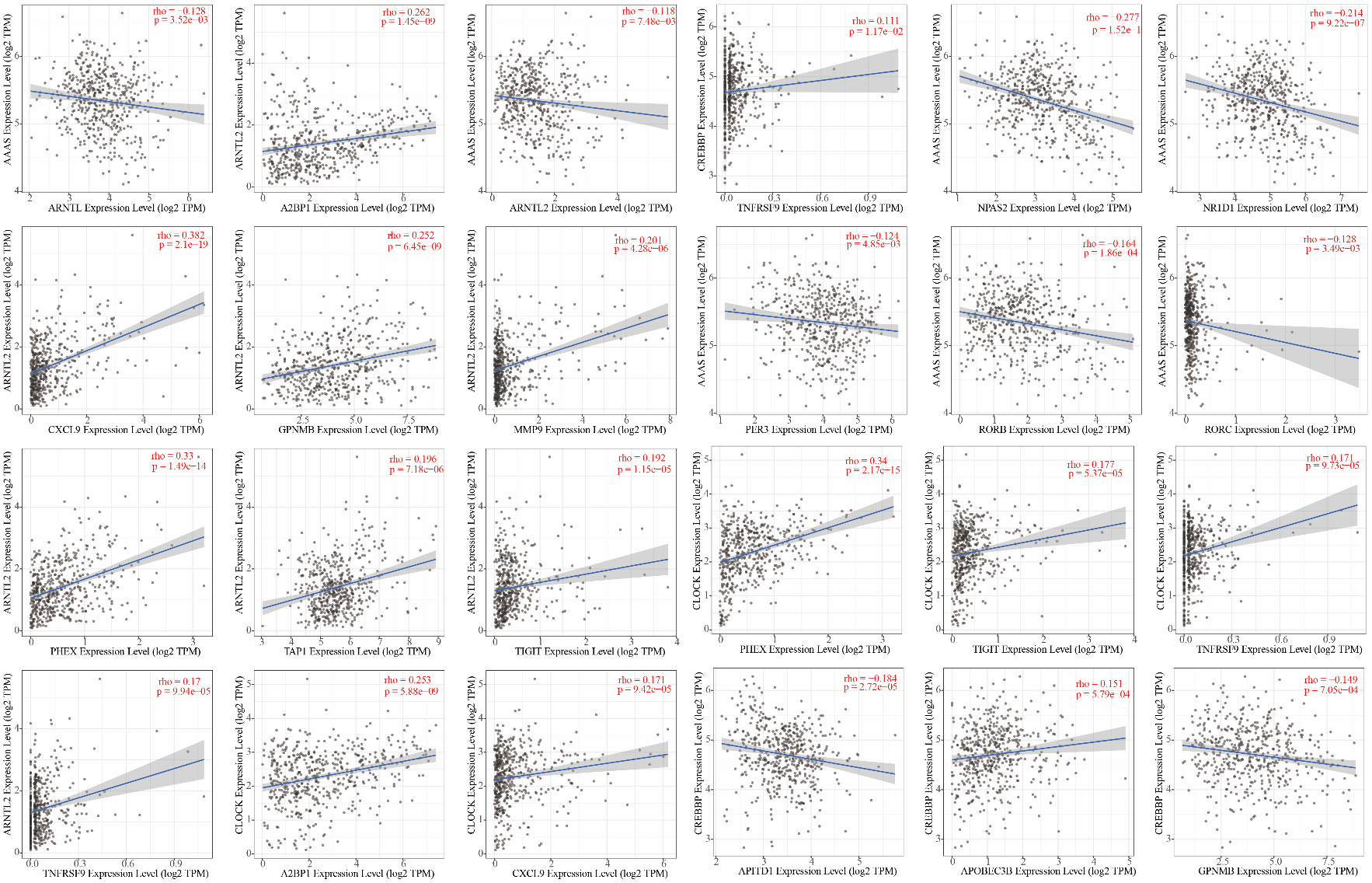


**Figure S6** Two-sample mendelian randomization (MR) results including SNP results (forest plots), funnel plots, leave-one-out analyses and scatter plots.


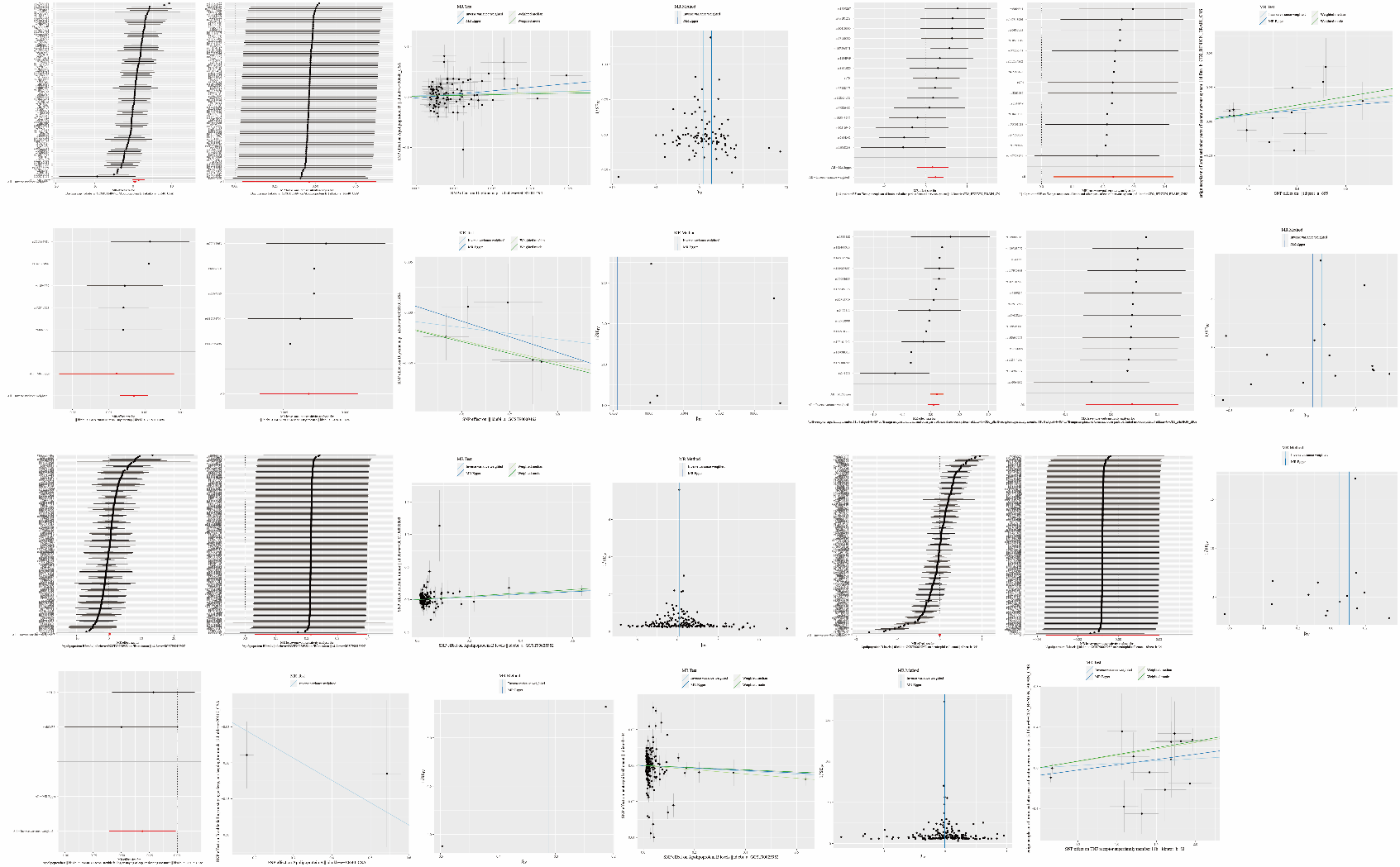

Supplement: Supplementary file 1 — Supplementary Figures. [file 41598_2024_67559_MOESM1_ESM.docx]
